# The cognitive and psychiatric subacute impairment in severe Covid-19

**DOI:** 10.1038/s41598-022-07559-9

**Published:** 2022-03-03

**Authors:** Pedro J. Serrano-Castro, Francisco J. Garzón-Maldonado, Ignacio Casado-Naranjo, Angela Ollero-Ortiz, Adolfo Mínguez-Castellanos, Mar Iglesias-Espinosa, Pablo Baena-Palomino, Violeta Sánchez-Sanchez, Rosa María Sánchez-Pérez, José Rubi-Callejon, José Carlos Estévez-María, Benito Galeano-Bilbao, Jesús Romero-Imbroda, Beatriz Sobrino, Carlos Arrabal-Gomez, Begoña Oliver-Martos, Luis Muñoz-Becerra, Nerea Requena, María del Mar González Álvarez de Sotomayor, Guillermo Estivill-Torrus, Juan Suarez, Nicolas Lundahl Ciano-Petersen, Gracia Pons-Pons, Jose Antonio Reyes-Bueno, Pablo Cabezudo-Garcia, Maria José Aguilar-Castillo, Carlos De la Cruz Cosme, María Duque-Holguera, Eva Cuartero-Rodriguez, Rosa María Vilches-Carrillo, Ismael Carrera-Muñoz, Cristóbal Carnero-Pardo, Teresa Ramirez-Garcia, Juan Manuel Oropesa, Ana Dominguez-Mayoral, Nazaret Pelaez-Viñas, Lucia Valiente, Fernando Rodríguez de Fonseca

**Affiliations:** 1 Neurology Service, Regional University Hospital of Malaga, Malaga, Spain; 2 Neurology Service, Virgen de la Victoria University Hospital, Malaga, Spain; 3 Neurology Service, University Hospital of Cáceres, Cáceres, Spain; 4 Neurology Service, Valme University Hospital, Seville, Spain; 5 Neurology Service, Virgen de las Nieves University Hospital, Granada, Spain; 6 Neurology Service, Torrecárdenas University Hospital, Almería, Spain; 7 Neurology Service, Juan Ramón Jiménez University Hospital, Huelva, Spain; 8 Neurology Service, Virgen Macarena University Hospital, Seville, Spain; 9 Neurology Service, University Hospital of Alicante, Alicante, Spain; 10 Neurology Unit, Internal Medicine Service, Hospital del Poniente de Almería, El Ejido (Almería), Spain; 11 Neurology Service, Reina Sofia University Hospital, Cordoba, Spain; 12 Neurology Service, Puerta del Mar University Hospital, Cadiz, Spain; 13 Neurology Service, Hospital Quirón-Salud Málaga, Malaga, Spain; 14 Infectious Diseases Service, Regional University Hospital of Malaga, Malaga, Spain; 15 Biotechnology Unit, Regional University Hospital of Malaga, Malaga, Spain; 16 Neuropsychopharmacology Group, Institute of Biomedical Research of Malaga (IBIMA), Malaga, Spain; 17 Neuroimmunology and Neuroinflamation Group, Institute of Biomedical Research of Malaga (IBIMA), Malaga, Spain; 18 Andalusian Network for Clinical and Translational Research in Neurology (NEURO-RECA), Malaga, Spain; 19 Fydian Neurocenter, Granada, Spain; 20 Biomedical Network Research Center for Neurodegenerative Diseases (CIBERNED), Madrid, Spain; 21 University Institute of Biosanitary Research of Extremadura (INUBE), Cáceres, Spain; 22 Institute of Biosanitary Research of Granada (Ibs. GRANADA), Granada, Spain; 23 Neurology Department, Instituto IBIMA, Hospital Regional Universitario de Málaga (España), Avda Carlos-Haya S/N, 4a planta, Malaga, Spain; 24 Neuropsychopharmacology Group, Instituto IBIMA, Hospital Regional Universitario de Málaga (España), Avda Carlos-Haya S/N, Malaga, Spain

**Keywords:** Cognitive neuroscience, Diseases of the nervous system, Neuroimmunology, Central nervous system infections, Dementia, Neurodegenerative diseases

## Abstract

Neurologic impairment persisting months after acute severe SARS-CoV-2 infection has been described because of several pathogenic mechanisms, including persistent systemic inflammation. The objective of this study is to analyze the selective involvement of the different cognitive domains and the existence of related biomarkers. Cross-sectional multicentric study of patients who survived severe infection with SARS-CoV-2 consecutively recruited between 90 and 120 days after hospital discharge. All patients underwent an exhaustive study of cognitive functions as well as plasma determination of pro-inflammatory, neurotrophic factors and light-chain neurofilaments. A principal component analysis extracted the main independent characteristics of the syndrome. 152 patients were recruited. The results of our study preferential involvement of episodic and working memory, executive functions, and attention and relatively less affectation of other cortical functions. In addition, anxiety and depression pictures are constant in our cohort. Several plasma chemokines concentrations were elevated compared with both, a non-SARS-Cov2 infected cohort of neurological outpatients or a control healthy general population. Severe Covid-19 patients can develop an *amnesic and dysexecutive syndrome* with neuropsychiatric manifestations. We do not know if the deficits detected can persist in the long term and if this can trigger or accelerate the onset of neurodegenerative diseases.

## Introduction

In December 2019, a new coronavirus emerged as a pathogen in the Chinese city of Wuhan, causing severe acute respiratory syndrome (SARS) of high lethality^1^. SARS-CoV-2 spread rapidly throughout the world, and the WHO declared the disease caused by this global virus a pandemic in March 2020. At the time of writing this article, more than 304 million people worldwide had been infected with SARS-CoV-2, resulting in the death of more than 5.5 million individuals^2^. Despite the acute consequences of SARS-CoV-2 infection, patients have described lingering symptomatology after the infection, a condition now called Long Covid.

Both, neurological impairment and psychiatric symptoms in this disease has been proven, both in the acute and subacute phases^3–7^. There are several mechanisms by which this neurological dysfunction can occurs such as direct viral invasion, indirect effects of peripheral inflammation mediated by alteration in blood–brain barrier (BBB) function, peripheral organ dysfunction (lung, kidney, and liver), cerebrovascular endothelial injury^8,9^ and others.

Necropsic studies have proven some neuroinvasive capacity of SARS-CoV-2^7,10,11^. This virus may enter the brain through three potential mechanisms: transsynaptic spread from the olfactory bulb following intranasal exposure, migration across the BBB through endothelial cell infection, and migration following disruption of the BBB from resulting inflammation^12–14^. Pathologic studies have found a high prevalence of SARS-CoV2 RNA and surface protein structures in olfactory mucosa^11^. Further evaluation identifying surface proteins of SARS-CoV2 through electron microscopy documented that endothelial cells in these regions were the primary target of infection^11^.

Furthermore, the relationship between viral neuroinvasive infections and neurodegenerative diseases (NDDs) has been described^15–19^, with preferent injury to the hippocampus and other regions of the temporal and frontal lobes related to cognition^20,21^_._

Secondly, a loss of function of the BBB can occur in situations of persistent systemic inflammation such as that occurring in SARS-Cov2 infection, making possible for immune molecules and cells to enter the CNS^22–24^. BBB dysfunction is pathogenically related to cognitive disorders related to ageing^25^, and neurodegenerative diseases or chronic psychiatric diseases, specially depression^26^. Interestingly, endothelial cells are key to the functional integrity of the BBB, and endothelial injury is a recognized element in the pathophysiology of SARS-CoV-2 infection^11,26^.

Third, peripheral tissue injury typical of serious infections, such as severe COVID-19 can generate danger-associated molecular patterns (DAMPs) or pathogen-associated molecular patterns (PAMPs), enough for acting on CNS-specific receptors, leading to microglial activation and, ultimately, pyroptosis or neuronal death of neuroinflammatory origin^27,28^. In this sense, the most relevant study on neuropathology of surviving patients of COVID-19 infection shows the microglial activation as one of the most important findings, especially in the hippocampus and brainstem, with the cognitive consequences that these findings may entail^29^. Moreover, a recent report identified the presence of serum neurofilament light chain, a marker of axonal damage, as a predictor of worse clinical outcomes in acute COVID-19 patients^30^, supporting the notion of. COVID-19-associated neuronal loss.

Finally, some other mechanisms have been suggested to justify brain damage. Some authors defend the pathogenic role of anti-SARS-Cov2 antibodies in CSF in the genesis of encephalopathy in the absence of markers of neuroinflammation^31^. On the other hand, recently it has been speculated that the role that choroid plexus dysfunction secondary to virus infection may play in brain damage^32^.

Cognitive and neuropsychiatric impairment persisting months after acute SARS-CoV-2 infection has been described^33–40^. A recent meta-analysis analyzed 81 studies on cognitive function in patients surviving COVID-19 infection showing that a fifth of these individuals exhibited cognitive impairment 12 or more weeks following confirmed infection^35^. Furthermore, in contradistinction to other persistent symptoms which may be self-limiting (e.g., anosmia) cognitive impairment appear to endure and may potentially worsen over time in susceptible individuals^36^. Another very recent systematic review of 39 studies showed that there are cognitive impairments in 15%, as well as anxiety (34%) and depression (32%) in patients with post-COVID syndrome. Psychiatric symptoms might be related to viral-induced neuroinflammation. In this regard, persisting changes in chemokines have been detected in both, mild depression^41^ and convalescent Covid patients^42^. Finally, decreased quality of life was reported by 57% of these patients^37^.

Some studies have analyzed the neuropsychological profile of the post-acute phase of COVID-19 infection. For example, Zhou et al.^38^ assessed cognitive function 3 weeks after hospital discharge of 29 patients with COVID-19, reporting a dysfunction in the sustained attention domain and a correlation between serum C-reactive protein (CRP) level and reaction time.

On the other hand, an Italian study on 38 hospitalized patients for SARS-CoV-2 infection in non-intensive COVID units showed that 42% had a slowing of cognitive processing speed and about 20% showed long-term verbal and spatial memory dysfunctions five months after hospital discharge^39^. Some studies have shown deficits in more specific cognitive domains. So, Impaired executive functions were observed in 33% of severe COVID-19 patients after hospital discharge in a pioneer French study by Helms et al.^40^.

However, we lack detailed studies that analyze from a more comprehensive perspective the selective involvement of the different cognitive domains using specifically designed tests, it impacts on quality of life and the possible existence of related biomarkers, including chemokines and neurofilaments, which have been involved in both, depression, and cognitive impairment. This is the goal of our study on a hospital cohort of survivors of a severe SARS-Cov-2 infection.

## Material and methods

### Design

Cross-sectional study of consecutive patients who survived severe infection with SARS-CoV-2; The clinical treatment of the patients was performed according to the routine clinical care based on the criteria of the attending physician of each patient.

#### Inclusion criteria

- At least one positive PCR test (oropharyngeal swab) for SARS-CoV-2 infection.
- Respiratory failure with criteria for hospital admission; radiological criteria for lung disease (chest CT scan/X-ray with bilateral ground-glass opacities).
- More than 90 days and less than 120 days since hospital discharge.

#### Exclusion criteria

- Cognitive impairment with a global deterioration scale (GDS) score of 4 or higher.
- Motor, sensorial, or intellectual disability or illiteracy that prevented performing neuropsychological tests.

### Recruitment

Patients were consecutively recruited from 13 neurology services in Spain during the first wave of pandemic through a retrospective review of patients with hospital admission for severe Covid-19. For comparison purposes on circulating plasma chemokines and growth factors, plasma samples from two non-SARS-Cov-2 infected control groups were used. The first group was a cohort of neurological patients (n = 46, mean age 71 y.o., SD 10.1, 17 males and 29 females) 60% affected with mild cognitive impairment (Mean MOCA score 18.5, SD 7.6). The second control group was a healthy general population from the National ADN biobank of Salamanca (n = 40, mean age 52.2 y.o., SD 2.3, 20 males and 20 females). Both groups were recruited for a different project and its use was approved by the ethical committee of Regional University Hospital of Málaga (RUHM).

#### Study variables

##### Retrospective data collected during admission period

- *Clinical data:* Age, sex, length of hospital stays, comorbidities, symptoms related to SARS, neurological symptoms during admission and anti-COVID drug treatment.
- *Analytical data:* Complete blood count, serum electrolytes, total protein, C-reactive protein, D-dimer, creatine kinase (CK), lactate dehydrogenase (LDH), transaminases, blood urea nitrogen (BUN), creatinine and ferritin.

##### Data collected during the visit (90–120 days after hospital discharge)

- *Neuropsychological study protocol* Global Cognition was studied through the Montreal Cognitive Assessment (MoCA) for dementia.

Memory was evaluated using the Spanish version of the California Verbal Learning Test (CVLT) also named Test de Aprendizaje Verbal España-Complutense (TAVEC) and Free and Cued Selective Reminding Test (FCSRT) for verbal episodic memory, Boston Naming Test (BNT) for denomination capacity, Rey Complex Figure Test (RCFT) for visuospatial episodic memory, and Digit Retention Test (DRT) of Wechsler Adult Intelligence Scale (WAIS) for working memory and memory reserve.

Executive function (RCFT, Trail Making Test Time B (TMT-B) and the Verbal Fluency Test (FAS)) and attention (TMT-A), were also evaluated.

Psychiatric impairment was evaluated using the State-Trait Anxiety Inventory (STAI) and Beck Depression Inventory II (BDI-II) tests.

All tests have been previously standardized for age, sex, and educational level for the Spanish population. Specifically, FCSRT, BNT, RCFT, FAS, DRT (verbal span) and TMT-A, and B were standardized within the framework of the NEURONORMA project^43–46^. The rest of the tests have been standardized for age, sex and education level when possible according with their respective published normative values: TAVEC^47^, MoCA^48^, STAI^49^ and BDI^50^.

An estimate of quality of life was made with the EuroQol 5D test (EQ-5D), also validated for Spanish population^51^.

##### Analytical study protocol

- *Basic screening:* The same as that collected during admission.
- *Plasma chemokines and growth factors:* MIP-1alpha/CCL3 (Macrophage inflammatory proteine-1-alpha), SDF-1 (Stromal cell-derived factor 1), Fractalkine/CX3CL1, Eotaxin 1, BDNF (Brain derived neurotrophic factor), VEGF (Vascular Endothelial Growth Factor) and MCP-1/CCL2 (Monocyte Chemotactic Protein-1) levels were determined using the Luminex™ xMAP technology platform. All samples, control groups and COVID patients were measured in the same plates for avoiding interassay variability^41^.
- *Serum neurofilaments:* Light Chain Neurofilament (NFL) levels, recently implicated in prognosis of severe COVID-19^30^, were determined using a digital enzyme immunoassay and the SIMOA HD1 Analyser platform.

### Interpretation of the tests

To reduce the impact of the absence of a cognitive function study prior to infection on the correct interpretation of our results, we have used tests with normative values for the Spanish population. To categorize each patient individual result in normal or abnormal values we used cut-off points stratified by age, sex, and educative level. Abnormal values were defined by ± 1 SD (± 1Z) of the mean for the reference group of age, sex, and educational level. This criterion was chosen instead of others, such as ± 1.5 SD, to increase the sensitivity of the deviation from the mean when categorizing the test results; we did not intend to categorize the results as “healthy” or “pathological” but simply detect deviations from normal state.

See the supplementary material for a list of the tests (Supplementary Table S2) and for an explanation of the protocol used for neuropsychological evaluations (Supplementary Box 1).

### Statistical study

All clinical data, laboratory variables and diagnostic tests were entered into a database for analysis using statistical software IBM SPSS version 21.0. For numerical data, the normality of the distribution of the data was determined by the Kolmogorov–Smirnov and Shapiro Wilk test. Data calculated as percentages were analysed using the chi-squared test. For the data expressed as the mean ± standard deviation, Student’s T test or the Mann–Whitney/Kruskal Wallis test were used depending on the normality of the sample. In case of normality of the distribution, family wise correction tests (Bonferroni) were used. When nonparametric Kruskal–Wallis test was used, Dunn’s test correction for multiple comparisons was used for controlling errors.

To determine the main components of our database, principal components analysis (PCA) was used; the analysis included quantitative variables of the neuropsychological test as well as age, length of hospital stay, and the analytical parameters found to be pathological during hospital admission since were considered interesting to define the characteristics of the syndrome. The Bartlett sphericity test and the Kayser–Meyer–Olkin (KMO) sample adequacy test were applied to demonstrate the adequacy of this type of analysis for our sample.

Correlations of the isolated components were analysed using Pearson’s R for continuous and normally distributed data and using Spearman’s rho for nonnormally distributed data.

Last, linear regression analysis of isolated components was performed using the score on the EQ5 quality of life scale as the dependent variable.

The missing data were excluded from statistical analysis except in the PCA, in which they were replaced by the mean.

### Ethical considerations

This study was approved by the Ethics and Clinical Research Committee of the RUHM (PEIBA internal code: 0894-N-20). Each participant or legal representative signed an informed consent form after receiving a complete description of the study and being given the opportunity to ask any questions. The process of obtaining informed consent adhered to the principles of the Declaration of Helsinki of the World Medical Association.

## Results

A total of 152 patients infected with SARS-CoV-2 who met all inclusion criteria and none of the exclusion criteria were recruited. The group was composed of 46 patients with long-term depressive symptoms, 25 with a history of stroke (16 territorial and 9 lacunar), 11 with chronic anxiety symptoms, 6 with Parkinson’s disease, 6 with subjective memory failure (with GDS < 4), 3 with Multiple Sclerosis, 3 with non-lesional focal epilepsy, 1 with Guillain–Barré syndrome and 11 with another chronic neurologic conditions without dementia. In this sense, we must consider that our cohort is composed mainly of patients with neurological or psychiatric vulnerability but without cognitive impairment, as required by the inclusion criteria.

The epidemiological data, symptoms during admission and specific treatments received during admission are provided in Table S1.

The means for the analytical variables assessed during admission were within the normal range of our laboratory, except the following:

- D-dimer: mean value, 1266.02 ng/ml (SD: 1969);
- Ferritin: mean value, 703.53 mcg/l (SD: 662.22) and;
- C-reactive protein (CRP): mean value, 94.49 mg/l (SD: 85.45).

Compared with the values obtained during the study visit (90–120 days after hospital discharge), the ferritin values were found in the normal range for our laboratory**,** while the D-dimer (586.36 ng/ml; SD = 683.75) and CRP (6.47 mg/ml; SD = 16.15) values remained slightly elevated, although their values had decreased substantially.

All analytical test results are available as supplementary material to this article (Supplementary Table S3).

Table 1 provides the psychopathological evaluation data and total MoCA test score as well as the scores for the 7 subdomains for the sample.

**Table 1:** Results of the psychopathological assessment (BDI, Stai tests) and MoCA test scores in the global sample.

	N	Average direct score (± SD)	Maximum score	Average scaled score	N (%) ≤ 1Z
BDI	135	14.95 (± 10.73)			37 (27.40%)
STAI state	138	23.79 (± 10.98)			49 (35.56%)
STAI trait	138	24.18 (± 11.18)			41 (29.49%)
Global MoCA score	131	21.95 (± 5.70)	30	10.86 (± 8.85)	33 (25.2%)
Visuo-spatial and executive function	129	3.63 (± 1.49)	5		
Animal naming	129	2.78 (± 0.53)	3		
Attention	129	4.23 (± 1.66)	6		
Language	129	2.07 (± 1.10)	3		
Abstraction	129	1.33 (± 0.70)	2		
Delayed recall	129	1.94 (± 1.69)	5		
Orientation	129	5.62 (± 0.92)	6		

Abnormal values are considered if they are equal to or less than 1 SD below the value corresponding to their standardized group by age, sex and educational level.

Table 2 provides the results of the cognitive exhaustive evaluation test used for the sample.

**Table 2:** Complete cognitive evaluation of the cohort.

	N	Average direct score (± SD)	Average scaled score (± SD)	Z (± SD)	N (%) ≤ 1Z
**TAVEC (verbal episodic memory)**					
TAVEC learning	118	35.91 (± 15.02)		− 0.87 (± 1.54)	49 (41.5%)
TAVEC short-term free memory	118	8.01 (± 5.55)		− 0.30 (± 1.74)	39 (33.3%)
TAVEC recall with short-term keys	118	8.77 (± 3.7)		− 0.55 (± 1.17)	41 (34.7%)
TAVEC long-term free memory	117	7.53 (± 4.2)		− 0.61 (± 1.27)	41 (35.0%)
TAVEC recall with long-term keys	117	8.77 (± 3.87)		− 0.59 (± 1.27)	45 (38.5%)
TAVEC recognition	117	13.36 (± 3.2)		− 0.26 (± 1.60)	24 (20.4%)
**BNT (denomination)**					
BNT	141	12.30 (± 3.13)		0.06 (± 1.37)	24 (17.0%)
**RCFT (visuospatial episodic memory/executive function)**					
RCFT time copy	123	242.11 (± 127.66)	11.67 (± 4.72)	0.56 (± 1.57)	19 (17.4%)
RCFT copy direct score	109	28.73 (± 9.42)	10.46 (± 3.51)	0.15 (± 1.17)	18 (14.7%)
RCFT memory direct score	101	11.29 (± 8.47)	8.26 (± 3.69)	− 0.58 (± 1.23)	40 (39.6%)
**TMT (attention/executive function)**					
TMT time A (attention)	114	94.96 (± 80.83)	7.71 (± 3.7)	− 0.76 (± 1.24)	39 (34.2%)
TMT errors A	108	0.46 (± 1.23)			
TMT time B (executive function)	91	182.25 (± 141.33)	8.93 (± 3.15)	− 0.36 (± 1.05)	28 (31.1%)
TMT errors B	87	1.87			
PMR as Spanish version of FAS (executive function* and verbal fluency**)					
FAS-P	142	10.00 (± 5.27)	8.06 (± 3.40)	− 0.48 (± 1.01)	47 (33.1%)
FAS-M	142	8.49 (± 4.92)	8.56 (± 3.53)	− 0.28 (± 1.07)	38 (26.8%)
FAS-R	142	8.43 (± 4.73)	8.44 (± 4.74)	− 0.16 (± 0.84)	30 (21.1%)
FAS animals	141	13.54 (± 5.69)	7.30 (± 3.12)	− 0.70 (± 0.96)	62 (43.7%)
FAS vegetables	142	14.00 (± 5.27)	8.63 (± 3.32)	− 0.80 (± 1.08)	69 (48.6%)
FAS kitchens	142	12.26 (± 4.45)	9.99 (± 3.61)	− 0.39 (± 1.19)	47 (33.1%)
**DRT (WAIS) (working memory/memory reserve)**					
WAIS direct span	121	4.95 (± 7.54)	8.11 (± 3.64)	− 0.63 (± 1.21)	44 (36.7%)
WAIS reverse span	121	3.25 (± 1.38)	11.32 (± 17.26)	− 0.05 (± 1.15)	32 (26.4%)
**FCSRT (verbal episodic memory)**					
FCSRT free memory	128	19.53 (± 9.07)	9.23 (± 3.54)	− 0.25 (± 1.18)	40 (31.25%)
FCSRT cued memory	127	17.11 (± 7.16)			32 (26.3%)
FCSRT total	127	35.76 (± 11.04)	10.17 (± 4.19)	0.06 (± 1.39)	32 (25.2%)
FCSRT delayed	127	6.74 (± 3.95)	9.10 (± 3.54)	− 0.30 (± 1.28)	39 (30.7%)
FCSRT total delayed	127	11.10 (± 4.77)	9.50 (± 4.92)	− 0.16 (± 1.64)	42 (33.1%)
**Quality of life**					
EQ-5D	141	62.94 (± 21.84)			

**Figure 1:**
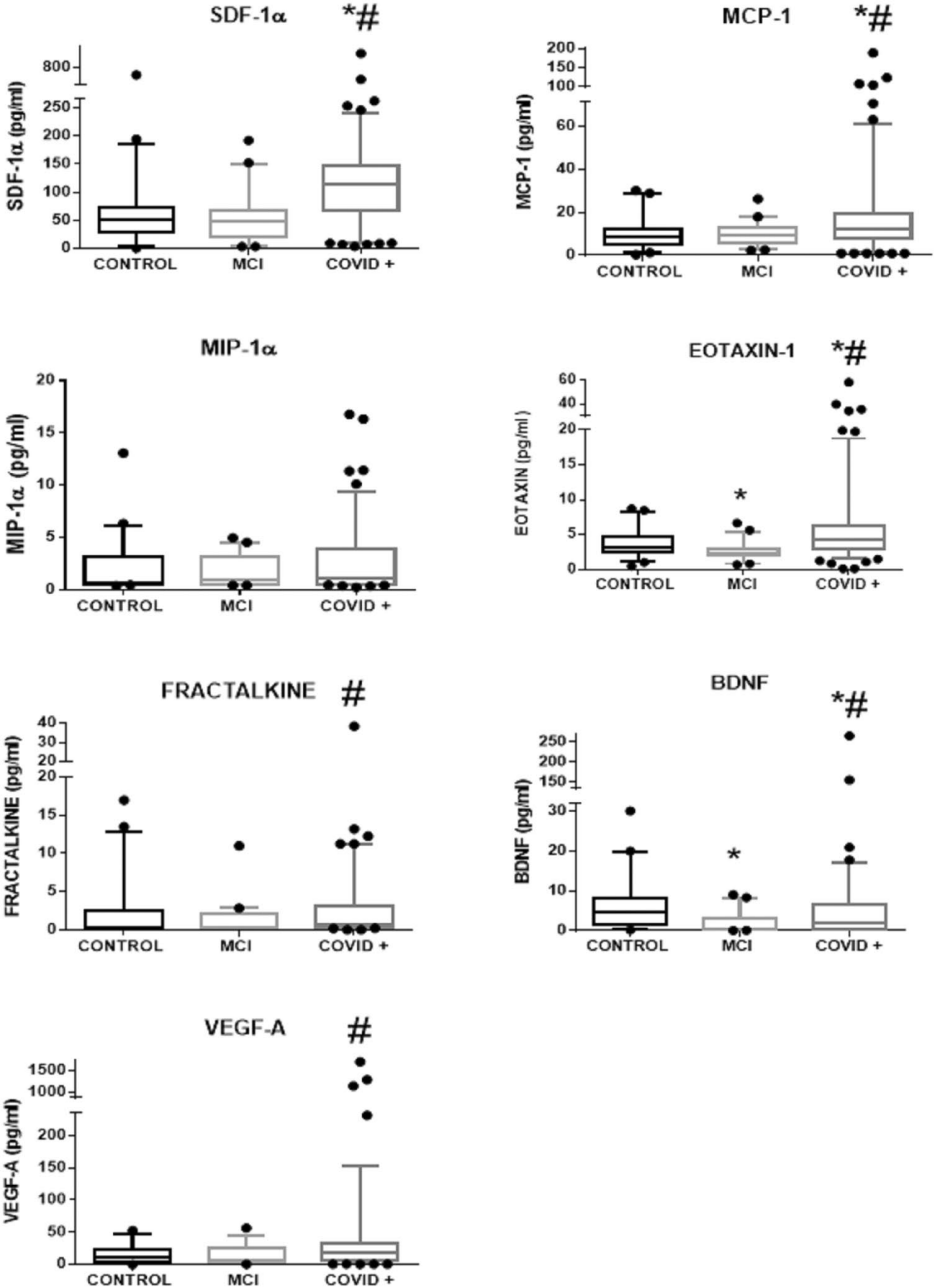
Plasma values of several chemokines and growth factors in control subjects (n = 45), mild cognitive impairment patients (MCI, n = 41) and COVID-19 patients (COVID+, n = 128) 3–4 months after hospital discharge. Kruskal–Wallis Analysis. *p < 0.01 versus control group. ^#^p < 0.01 versus MCI group. Data in boxplots are means and 5–95 confidence intervals.

Figure 1 provides the data for chemokines and growth factors in general population, mild cognitive impairment control group and COVID patients.

Supplementary Table S4 provides the numerical data for chemokines, growth factors and NFL.

**Figure 2:**
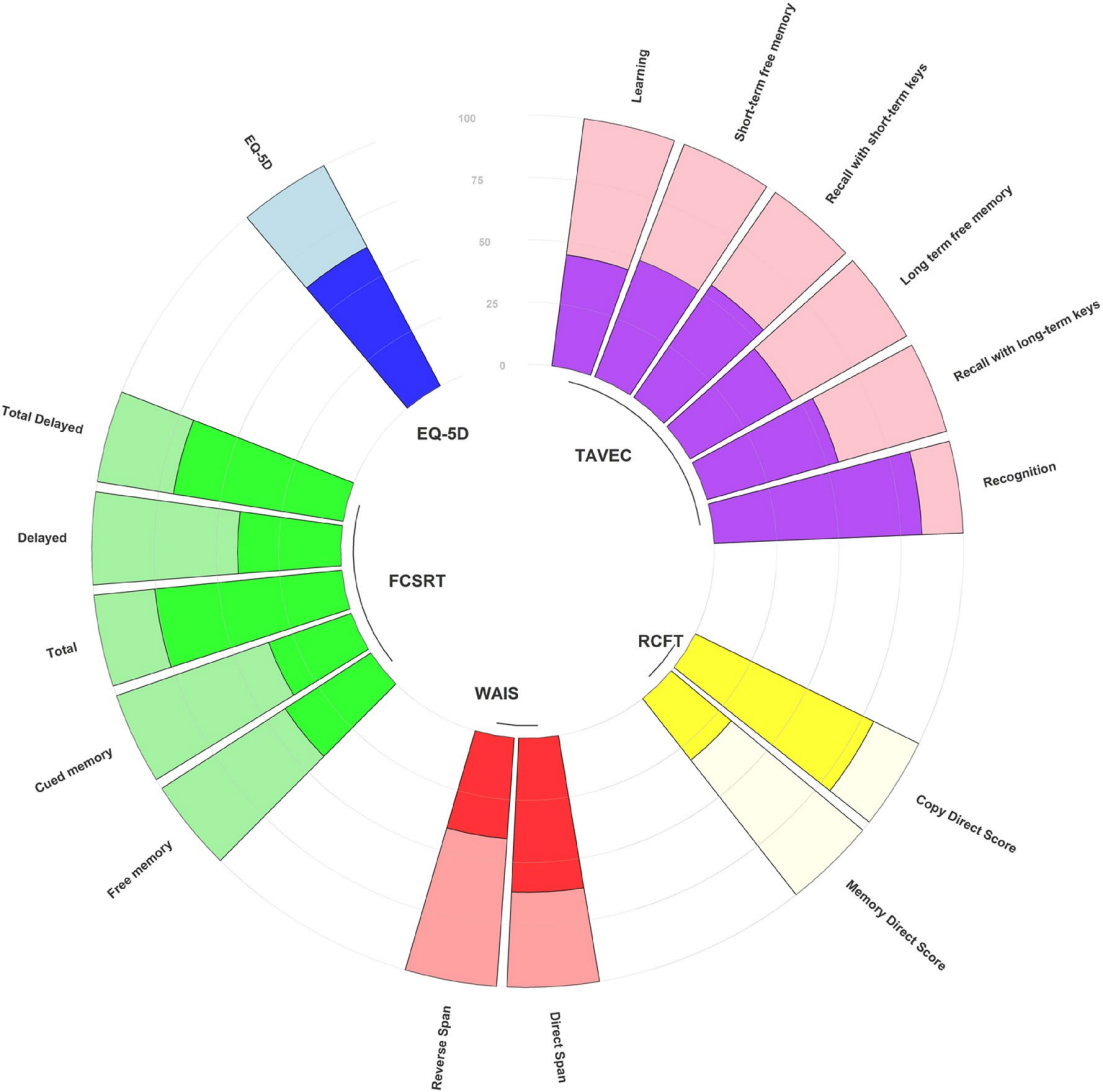
Graphical representation of the profile of the main test used in our study comparing the average vs maximum score.

For a graphical representation of the cognitive deficits detected in our sample the results are presented as a function of the percentage of the maximum score obtained on each test and as the total score (Fig. 2).

### Principal components analysis

To reduce the number of variables, we performed PCA, in which we included the following quantitative variables: age, length of stay, pathological analytical variables during admission (ferritin and D-dimer) and numerical variables corresponding to the cognitive and neuropsychiatric test results. Six components capable of explaining 55.34% of the variance were identified. The KMO value was 0.854, and the Bartlett sphericity test indicated a significance of < 0.0001, confirming the power and adequacy of the analysis.

The rotated components matrix and the explained variance table are provided as Supplementary Material (Supplementary Tables S5, S6, respectively). When a variable showed correlation with more than one Component was assigned to the one with the highest Factorial load. Factorial loads less than 0.5 were not considered. Finally, to choose the number of components we considered the total cumulative variance explained and their coherence from the clinical point of view.

After PCA, the following 6 independent components were identified as the cognitive and psychopathological areas affected.

#### Components 1 and 5

These 2 components are grouped because they integrate *variables related to episodic memory*, that is, memory related to vital events. Component 1 includes some scores of the TAVEC test as well as the free memory score of the FCSRT test, which primarily evaluates episodic verbal memory. Furthermore, Component 5 includes the other scores for the FCSRT test (cued and delayed recall).

Impairment in *episodic verbal memory* was observed in 34.7% (for TAVEC short term free memory) to 38.5% (for TAVEC Recall with long-term keys) of our patients and constitutes a specific element of this syndrome. Additionally, *working memory* measured through the Digit Retention Test (DRT) (WAIS-IV), was affected in 26.4–36.7% of the sample. Other types of memory such as *semantic memory*, explored through the BNT seem less affected.

#### Component 2

This component includes variables related to global cognitive function and visuo-spatial abilities, such as the overall MoCA score and subdomain scores, except for orientation and animal naming (which are integrated in Component 4), and the FCSR direct copy and memory scores, which both measure visuospatial and executive function.

The mean overall MoCA score in our sample was 21.95 (± 5.70) points. Deficits were identified by low scores in delayed recall and, to a lesser extent, in attention, abstraction and language.

#### Component 3

Component 3 includes variables related to *executive functions* and *verbal fluency*. The impairment of *executive functions* was substantial in our patients as shown by the scores of the FAS animals (43.7% abnormal), FAS vegetables (48.6% abnormal) and FAS kitchen (33.1% abnormal) tests, more related to executive functions. We attribute this result to a failure in executive functions reinforcing the idea that frontal lobe dysfunction is frequent in post-Covid19 syndrome.

#### Component 4

The component includes *variables related to attention*, especially TMTs A, and *orientation and naming* (subtests of the MoCA). The scores obtained were abnormal for the 34.2% of the sample for the TMTs A.

#### Component 6

Depression and anxiety related variables. The BDI-II scores for our sample correspond to mild depression. Overall, 27.4% of patients had a BDI score equal to or greater than 20 points, which indicates a clinical diagnosis of moderate or severe depression^52^. A total of 35.56% of the total sample had state STAI-State scores compatible with state anxiety.

### Correlation with quality of life

A regression analysis of the identified components was performed using quality of life as the dependent variable; the results are provided in Supplementary Tables S7–S9). A Durbin-Watson test value less than 2 ensures that the factors are not autocorrelated. The result of the analysis showed that components 2 (global cognition/executive functions) and 6 (impairment of the neuropsychiatric area) explained the variable quality of life with high significance.

### Plasma concentration of proinflammatory chemokines and growth factors (Fig. 1)

We selected five chemokines and two growth factors that have been related to neuroinflammation and cognitive impairment/neurodegeneration previously^41,53–57^. Kruskal–Wallis analysis show that the chemokines SDF-1a (H = 7.3. p < 0.001), MCP-1 (H = 14.1 p < 0.01) and Eotaxin-1 (H = 37.5. p < 0.001) were elevated in post-Covid patients, as well as the trophic factor BDNF (H = 28.7. p < 0.001), when compared with both control groups. In addition, Fractalkine (H = 14.0. p < 0.01), and VEGF-A (H = 11.1. p < 0.01) were elevated when compared only with the MCI cohort. MIP1-A was equal among groups (H = 4.9 p = 0.1, nonsignificant). These results suggest a pro-inflammatory chronic stated derived of severe COVID-19 disease. Remarkably, the circulating pattern of chemokines and growth factors in postcovid patients was found to be different of that of non-infected age-matched patients attending the neurology department because of subjective memory deficits complaints (MCI cohort). Specifically, there were significant differences with higher levels of neuroinflammation markers in post-Covid patients for all determinations except for MIP-1α.

### Correlation with plasma proinflammatory factors and NFL

To identify plasma markers that could potentially be related to the main components of this Syndrome, a bivariate correlation analysis was performed using PCA components and the values obtained for each given plasma factor. The results are shown in Table 3.

**Table 3:** Bivariate correlation between identified components and chemokine levels.

Measured factor	N	Component 1 (episodic memory)	Component 2 (global cognition)	Component 3 (executive functions)	Component 4 (attention)	Component 5 (episodic memory)	Component 6 (depression and anxiety disorder)
MIP-1 (CCL3)	106	0.107 (0.276)	0.013 (0.892)	0.048 (0.64)	− 0.061 (0.535)	− 0.060 (0.538)	0.002 (0.983)
SDF-1 (CXCL12)	117	0.028 (0.763)	− 0.053 (0.571)	0.106 (0.254)	− 0.148 (0.112)	− 0.008 (0.931)	0.072 (0.440)
Fractalkine (CX3CL1)	104	0.008 (0.932)	− 0.016 (0.875)	− 0.157 (0.111)	− *0.318 (0.001)*	− 0.034 (0.735)	− 0.115 (0.247)
Eotaxine (CCL11)	120	0.032 (0.729)	− 0.032 (0.726)	− *0.226 (0.013)*	− 0.064 (0.486)	− 0.060 (0.516)	0.022 (0.814)
BDNF	105	0.117 (0.233)	0.050 (0.611)	− *0.213 (0.029)*	− 0.024 (0.806)	− 0.157 (0.109)	− 0.149 (0.129)
VEGF	108	0.050 (0.606)	0.011 (0.910)	− *0.241 (0.012)*	− *0.203 (0.035)*	− 0.131 (0.178)	− 0.031 (0.754)
MCP-1 (CCL2)	116	− 0.005 (0.960)	− 0.077 (0.412)	− 0.174 (0.062)	− 0.102 (0.277)	0.017 (0.855)	− 0.128 (0.169)
NFL	58	− *0.310 (0.018)*	− *0.297 (0.024)*	− *0.417 (0.001)*	0.101 (0.452)	− 0.049 (0.717)	− 0.079 (0.554)

In all cases we expressed Spearson’s Rho (p).

Significance values are given in italics.

### Correlation with clinical variables

In any case, some clinical variables during admission were retrospectively collected such as length of hospital admission or the presence of clinical data such as fever, dyspnea, anorexia, consciousness impairment, seizures, or anosmia. This allowed us to make an analysis of association and/or correlation with the results of the neuropsychological study.

There was no correlation of the duration of admission with the global score in the MoCA or with the values of the 6 independent components of the PCA when we explored the correlation with the Spearman Test (non-normal variable).

The dichotomous clinically related variables mentioned above also showed no correlation through non-parametric test with the overall MoCA score and with the PCA components except in these cases:

- Anorexia was significantly correlated with Component 6 (depression/anxiety; p = 0.005).
- Impaired consciousness during admission was significantly correlated with the global MoCA score (p = 0.019), Component 2 (p = 0.033), an issue that we consider logical, given that Component 2 includes the overall MoCA score and Component 6 (depression/anxiety; p = 0.039).

## Discussion

The results of our study show a pattern of cognitive impairment with some peculiarities. Thus, there is preferential involvement of episodic memory, working memory, executive functions, attention, and relatively less affectation of information processing speed, denomination, verbal fluency, and other cortical functions such as visuo-constructive ability (Table 2, Fig. 2). In addition, the detection of psychiatric affectation such as anxiety and depression pictures are constant in our cohort (Table 1). So, we could therefore refer to post-covid syndrome as an amnesic and dysexecutive syndrome with impaired attention and affective psychiatric comorbidity. This pattern can be typified as suggestive of fronto-subcortical involvement, although further follow up of cases, and additional cohort studies are needed to fully determine this assumption.

The results of our PCA allows us to discuss some features in more detail:

1. Preferential involvement of *episodic memory* (represented by Components 1 and 5) in post-Covid syndrome. This is a key finding of our study. Traditionally, this type of mnemonic impairment has been related to subcortical cognitive impairment and has been identified in other neuroinflammatory processes of viral origin, such as HIV-associated neurocognitive disorders (HANDs)^20,58^.
2. Moderate impairment of *global cognition* and *visuo-spatial functions* (Component 2): The scores of the tests included in this component was not very deficient. Our patients are preferably included in the spectrum of mild cognitive impairment (MCI) with relative respect for purely cortical functions such as visuospatial function.
3. Relevant impairment of *executive functions* (Component 3).These findings are consistent with published functional impairment data, especially in studies that evaluated brain positron emission tomography with fluorodeoxyglucose (FDG-PET) and demonstrated greater impairment in the amygdala, hippocampus, parahippocampal region and frontal lobes^59–61^, areas directly related to memory and executive functions.
4. Impairment of *attention, orientation*, and *nomination* (Component 4). This finding is commonly described in patients with inflammatory-based encephalopathies^62,63^. The two MoCA subdomains included in this component that were less affected: Animal naming and Orientation.
5. Relevant impairment of variables related to *Depression* and *Anxiety* (Component 6): The results of our study show that regardless of its origin, neuropsychiatric impairment is another of the essential elements of the syndrome.

PCA is a technique for reducing the dimensionality of datasets, increasing interpretability but at the same time minimizing information loss. It does so by creating new uncorrelated variables that successively maximize variance. For this reason, the fact that there is an independent dimension (Component 6) that encompasses the variables related to the psychiatric state, guarantees that these are variables not correlated with the rest of the Components, which encompass variables related to cognitive function.

Cognitive impairment associated with depression is clearly seen in severe depression^64^. Perhaps in the subgroup of patients in our cohort in which we found moderate or severe depression (27.4%) this finding could influence the results of cognitive function and thus should be interpreted. However, if we consider our entire cohort, the degree of depression found is mild (mean score on the BDI-II = 14.95). For this reason, the non-existence of correlation with cognitive variables is not surprising.

Since, over the course of the pandemic, the general population has been subjected to stressors derived from the social and economic impacts of the virus^33,65^ is difficult to separate the actual contribution of the biological factors highlighted in this article from other environmental factors.

Some studies have shown a relationship between severity parameters during the acute phase and cognitive impairment in the medium term^66,67^. Our series focuses on patients who have suffered severe infection. The inclusion criteria established the need to suffer respiratory failure with criteria for hospital admission and radiological data for lung disease (chest CT scan/X-ray with bilateral ground-glass opacities). From this point of view, the systemic involvement was quite homogeneous in our cohort. This explains that we have not found differences between the cognitive impairment of patients based on factors such as length of admission or other clinical parameters collected during admission.

Integrating all these results, we can state that the subacute neurological impairment in severe Covid-19 can be defined as a *global cerebral condition in the spectrum of MCI, characterized by a predominant deterioration of memory (especially, episodic and working memory), executive functions, attention, and neuropsychiatric impairment.*

As we mentioned in the Introduction, several studies have also analyzed the characteristics of the cognitive deficit detected in patients who survive infection by SARS-COV2^35–40^. In general, these studies suffer from a lack of systematization of the neuropsychological analysis carried out. A systematic review that included 12 studies with a total patient sample of near 1000 people show us that patients with recent SARS-CoV-2 infection appears to experience global cognitive impairment and selective deficits in memory, attention, and executive function, and in particular verbal fluency^68^. These results are consistent with ours. Nevertheless, the authors of this review conclude that novel studies with larger sample and more comprehensive cognitive analysis are needed.

Our results identify a pattern of subcortical deterioration with similarities to that described in cerebral small-vessel diseases with the predominant endothelial injury^69,70^. There are also semiological parallels with neuroinflammation-based encephalopathies^63,71,72^. Among the plasma factors studied herein, some, such as CRP and NFL, are correlated with endothelial injury^73^.

Although our cohort is quite homogeneous, it could be hypothesized whether there could be different manifestations based on the previous existence of a situation of greater cognitive or psychiatric vulnerability. To test this possibility, we performed a post-hoc analysis dividing the sample into patients with a history of neurological or psychiatric disease versus those who did not. The results showed that there were no differences between these subgroups, thus highlighting the homogeneous nature of our results. We present these data as supplementary material (Tables S10–S13).

Quality of life was directly correlated with the main components that measure global cognitive function, and neuropsychiatric impairment; in the latter case, there was an inverse correlation with high trait anxiety and state anxiety scores and depression. We propose that impairment in these domains most determine the quality of life of our patients.

Interestingly, the analysis of circulating chemokines and growth factors suggest that 3 months after discharge, COVID-19 patients have a persistent neuroinflammatory state. This activation appears to be derived only of the infection by SARS-Cov-2, and not being associated to age-associated cognitive impairment, since patients with MCI not infected displayed a clearly different set of plasma chemokine concentrations. The contribution of this chemokine-based inflammatory state can be linked to the presence of psychiatric disorders, specially depression. A recent work^41^ in mild depression in primary care setting suggested that chemokines such as SDF-1, MCP-1 and fractalkine are associated to a higher punctuation on the BDI scale, a finding that replicates for those three chemokines in our cohort of COVID-19 patients. However, the correlation analysis of the components with plasma chemokine proinflammatory factors found few significant correlations. Thus, the exact contribution of this pro-inflammatory biomarkers to the clinical phenotype of Long Covid patients remains to be determined, but the rapid normalization of these biomarkers after effective interventions against depression suggest that they can be used for monitoring affective improvement in COVID-19 patients.

In addition, there was an inverse correlation between NFL levels and the Components related to the measurement of episodic memory (Rho =  − 0.310; p = 0.018), global cognition (Rho =  − 0.297; p = 0.024) and to Executive functions (Rho =  − 0.417; p = 0.001). NFL are markers of neuronal destruction whose correlation with global cognition has been described in the literature^74^. Plasma NFL levels could be a robust biomarker of this syndrome, and a recent study revealed that the course of its plasma concentrations is useful for determining the severity and prognosis of acute SARS-Cov-2 infection demanding hospitalization^30^.

Vascular Endothelial Growth Factor (VEGF) showed inverse correlation with the variables included in Component 4, mainly related to Attention. VEGF has been linked to endothelial dysfunction which, as already mentioned, appears to be an element present in SARS-Cov2 infection^8^ and more specifically, with cognitive decline present in some diseases with a large vascular component, such as DM^57^. This finding reinforces our hypothesis that Post-Covid Neurologic Syndrome is intimately related to the typical vascular damage of Covid-19 disease.

In conclusion this Syndrome is a distinct condition that persists for at least 12 weeks after overcoming the acute phase of severe SARS-CoV-2 infection. The profile remains stable in different stratified populations based on cognitive vulnerability. Last, we identified biomarkers related to the main components of the syndrome. The possibility of any of them behaving as a prognostic biomarker and even as possible future therapeutic strategy development for Post-Covid Neurologic Syndrome should not be ruled out.

Our study provides a systematization of the neuropsychological battery used that allows us to draw the identifying characteristics of the cognitive impairment of the post-viral phase of SARS-Cov-2 infection. In a recent systematic review by Salamanna et al.^75^, on the other hand, the authors highlight the absence of studies especially directed against vulnerable population with severe Covid-19 infection, so our study fills that gap. Other relevant finding in our study is the identification of a circulating pattern of chemokines and growth factors different of that of non-infected age-matched patients with MCI. Specifically, there were significant differences with higher levels of neuroinflammation markers in post-Covid patients.

The main limitation of our study is the absence of an assessment of cognitive and neuropsychiatric function and of plasma markers prior to infection, preventing us from reliably measuring the impact of the infection. To minimize the impact of this fact on the interpretation of our results, we have used tests that have normalized values for the Spanish population, so that we can consider that the real control group in this study is the own general population stratified by age, sex, and educational level.

Another limitation is the absence of neuroradiological data, which may have hidden data referring to structural lesions (vg ischemic lesions) with influence on the cognitive profile of our patients. Finally, we recognize that there has been a variable quota of missing data regarding both neuropsychological assessment and chemokines, with the latter especially involved.

Some issues remain to be resolved. First, we do not know whether the deficits detected are transitory or persist long term. If long term, it is unknown whether the underlying neuroinflammatory phenomena can trigger NDDs in a manner analogous to what probably occurred during the 1918 “Spanish flu” pandemic^19^.

## Supplementary Information

Supplementary Information.

## Data Availability

All databases used during the preparation of this article are available to editor or reviewers if required and have been incorporated into the DRYAD repertoire (Dryad Home—Publish and Preserve your Data (https://datadryad.org/stash)).
