# Supplementary material for: The cognitive and psychiatric subacute impairment in severe Covid-19
Source: Sci Rep. 2022 Mar 3;12:3563. doi: 10.1038/s41598-022-07559-9 (PMC8894467; doi:10.1038/s41598-022-07559-9)
Supplement: Supplementary file 1 — Supplementary Information. [file 41598_2022_7559_MOESM1_ESM.docx]

**SUPPLEMENTARY MATERIAL**

**Title: The Cognitive and Psychiatric Subacute Impairment in severe Covid-19.**

Authors: Pedro J. Serrano-Castro^1,17,18#^, Francisco J Garzón-Maldonado^2,18^, Ignacio Casado-Naranjo^3,20,21^, Angela Ollero-Ortiz^4,18^, Adolfo Mínguez-Castellanos^5,18,22^, Mar Iglesias-Espinosa^6,18^, Pablo Baena-Palomino^7,18^, Violeta Sánchez-Sanchez^8,18^, Rosa Maria Sánchez-Pérez^9^, Jose Rubi-Callejon^10,18^, Jose Carlos Estévez-María^11,18^, Benito Galeano-Bilbao^12,18^, Jesús Romero-Imbroda^1,13,18^, Beatriz Sobrino^14^, Carlos Arrabal-Gomez^18^, Begoña Oliver-Martos^17,18^, Luis Muñoz-Becerra^1,17,18^, Nerea Requena^2,18^, Maria del Mar Gonzalez Alvarez de Sotomayor^1,17,18^, Guillermo Estivill-Torrus^1,17,18^, Juan Suarez^16,18^, Nicolas Lundahl Ciano-Petersen^1,17,18^, Gracia Pons-Pons^1,18^, Jose Antonio Reyes-Bueno^1,17,18^, Pablo Cabezudo-Garcia^1,17,18^, Maria José Aguilar-Castillo^15,18^, Carlos De la Cruz Cosme^2,18^, Maria Duque-Holguera^3^, Eva Cuartero-Rodriguez^4,18^, Rosa Maria Vilches-Carrillo^5,18^, Ismael Carrera-Muñoz^5,18^, Cristóbal Carnero-Pardo^19^, Teresa Ramirez-Garcia^6,18^, Juan Manuel Oropesa^7,18^, Ana Dominguez-Mayoral^8,18^, Nazaret Pelaez-Viñas^11,18^, Lucia Valiente^14^, Fernando Rodríguez de Fonseca^16,18,#^

| Epidemiological characteristics | | |
| --- | --- | --- |
|  | | |
| Sex (V. %) | | 70/152 (46.1%) |
| Age in Years (±SD) | | 69.09 (±11.37) |
| Duration of admission in days (±SD) | | 14.38 (±13.14) |
| Ethnicity | Caucasian | 150 (98.7%) |
|  | Latin | 2 (1.3%) |
| Level of study | Primary education | 73 (48.0%) |
|  | Secondary education | 34 (22.3%) |
|  | Higher education | 21 (13.8%) |
|  | No studies | 15 (9.8%) |
|  | Unknown | 9 (5.9%) |
| Marital status | As a couple | 99 (65.1%) |
|  | Single | 9 (5.9%) |
|  | Widower | 18 (11.8%) |
|  | Separate | 21 (13.8%) |
| Previous GDS | GDS1 | 108 (71.0%) |
|  | GDS2 | 35 (23.0%) |
|  | GDS3 | 7 (4.6%) |
| Comorbidities | HT | 95 (62.5%) |
|  | DM | 39 (25.6%) |
|  | Previous cerebrovascular disease | 47 (30.9%) |
|  | Cancer disease | 8 (5.2%) |
|  | Smoking | 15 (9.8%) |
|  | Regular alcohol consumption | 28 (18.4%) |
| General symptoms during admission | | |
| Fever (Temperature > 38ºC) | | 123 (80.9%) |
| Cough | | 107 (69%) |
| Dyspnea | | 90 (58.1%) |
| Anorexia | | 47 (30.3%) |
| Diarrhea | | 55 (35.5%) |
| Neurological symptoms during admission | | |
| Headache | | 34 (21.9%) |
| Loss of awareness | | 11 (7.1%) |
| Seizures | | 1 (0.6%) |
| Stroke | | 2 (1.2%) |
| PNS involvement | | 6 (3.9%) |
| Myalgias | | 17 (11%) |
| Anti-Covid19 treatments | | |
| Hydroxychloroquine | | 112 (72.3%) |
| Lopinavir/Ritonavir | | 69 (44.5%) |
| Others (Methylprednisolone. Anti-IL) | | 98 (64.4%) |

Table S1. Epidemiological and clinical data during admission. DM: Diabetes mellitus. GDS: Global Deterioration Scale. HT: Hypertension. PNS: Peripheral Nervous System.

|  | | **CLINICAL EVALUATION** | | |
| --- | --- | --- | --- | --- |
|  | | **Cognitive area** | **Time (minutes)** | **Type of Administration** |
| **Psychopathology** | *BDI-II* | Depression | 5-10 | Autoreport |
|  | *STAI* | Anxiety | 5-10 | Autoreport |
| **Cognitive Status** | MoCA | Screening cognitive decline | 10 | Clinical staff |
|  | *TAVEC* | Verbal episodic memory | 40 | Clinical staff |
|  | *BNT* | Semantic memory | 10 | Clinical staff |
|  | *RCFT* | Visospatial episodic memory and executive function | 10 | Clinical staff |
|  | *TMT A and B* | Attention and executive function | 5-10 | Clinical staff |
|  | *DRT (WAIS-IV)* | Working memory | 10 | Clinical staff |
|  | *FAS* | Executive function and language | 6 | Clinical staff |
|  | *FCSRT* | Verbal episodic memory | 25 | Clinical staff |
| **Quality of Life** | *EQ-5D* | Health status | 1-2 | Autoreport |

Table S2: Battery of Neuropsychological Test. NPI: Cummins Neuropsychiatric Inventory. BDI-II: Beck-II Depression Inventory. STAI: State-Trait Anxiety Questionnaire. MoCA: Montreal Cognitive Assessment. TAVEC: Spain-Complutense Verbal Learning Test. BNT: Boston Naming Test. RCFT: Complex Figure of Rey-Osterrieth. TMT: Trail Making Test. DRT (WAIS-IV): Digit Retention Test (Weschler Intelligence Scale for Adults-IV). FAS: Florida Association of School Psychologists. FCSRT: Free and Cued Selective Reminding Test. EQ-5D: EuroQol 5D.

|  | Hospital admission | | | | Visit (90-120 days after discharge) | | | |
| --- | --- | --- | --- | --- | --- | --- | --- | --- |
|  | N | Average (±SD) | Median | Interquartile Range | N | Average (±SD) | Median | Interquartile Range |
| **Glucose (mg/dl)** | 144 | **123.19 (±41.09)** | 114.00 | 33.25 | 100 | **107.19 (±29.10)** | 99.00 | 24.50 |
| **Creatinine (mg/dl)** | 145 | **1.10 (±0.82)** | 0.90 | 0.44 | 100 | **0.88 (±0.26)** | 0.30 | 0.30 |
| **Ferritin (ng/ml)** | 100 | **703.52 (±662.22)** | 525.00 | 734.25 | 84 | **113.61 (±124.99)** | 72.65 | 108.92 |
| **C Reactive Protein (mg/l)** | 144 | **94.49 (±85.45)** | 68.89 | 119.15 | 93 | **6.47 (±16.15)** | 2.90 | 3.40 |
| **D-Dimer (ng/ml)** | 138 | **1266.02 (±1969.52)** | 685.00 | 940.75 | 81 | **586.36 (±683.75)** | 381.00 | 400.00 |
| **CPK (U/l)** | 71 | **195.32 (±506.94)** | 71.00 | 95.60 | 45 | **99.73 (±98.64)** | 76.70 | 68.40 |
| **Urea (mg/dl)** | 138 | **44.15 (±32.90)** | 33.50 | 22.40 | 81 | **39.39 (±13.39)** | 36.00 | 17.75 |
| **Albumin (g/dl)** | 52 | **3.47 (±0.86)** | 3.30 | 0.79 | 75 | **4.28 (±0.67)** | 4.20 | 0.62 |
| **Erythrocytes (x10^6^/mcl)** | 129 | **4.49 (±0.61)** | 4.50 | 0.70 | 83 | **4.63 (±0.73)** | 4.59 | 0.78 |
| **Hemoglobin (g/dl)** | 144 | **13.12 (±1.65)** | 13.10 | 1.88 | 101 | **13.70 (±1.65)** | 13.55 | 2.23 |
| **Hematocrit (%)** | 142 | **39.98 (±4.96)** | 39.95 | 6.03 | 100 | **41.45 (±8.62)** | 42.40 | 6.70 |
| **VCM (Fl)** | 142 | **89.59 (±6.13)** | 89.65 | 7.08 | 101 | **93.50 (±5.77)** | 93.50 | 6.90 |
| **Platelets (x10^3^/mcl)** | 143 | **233.22 (±99.55)** | 214.00 | 129.00 | 101 | **233.58 (±70.02)** | 224.00 | 85.50 |
| **Leukocytes (x10^3^/mcl)** | 144 | **7.43 (±3.11)** | 6.84 | 3.45 | 101 | **6.24 (±1.77)** | 5.94 | 2.07 |
| **Neutrophils (x10^3^/mcl)** | 144 | **5.69 (±2.89)** | 5.09 | 3.20 | 100 | **3.55 (±1.28)** | 3.41 | 1.65 |
| **Lymphocytes (x10^3^/mcl)** | 144 | **1.24 (±1.31)** | 1.06 | 0.70 | 100 | **1.96 (±0.75)** | 1.92 | 0.94 |
| **Monocytes (x10^3^/mcl)** | 143 | **0.56 (±0.34)** | 0.51 | 0.41 | 100 | **0.52 (±0.20)** | 0.51 | 0.19 |
| **Eosinophils (x10^3^/mcl)** | 143 | **0.03 (±0.07)** | 0.01 | 0.04 | 101 | **0.22 (±0.69)** | 0.49 | 0.13 |
| **Basophiles (x10^3^/mcl)** | 143 | **0.03 (±0.09)** | 0.01 | 0.02 | 100 | **0.04 (±0.03)** | 0.04 | 0.03 |
| **Prothrombin Time (sec)** | 140 | **13.34 (±4.57)** | 12.40 | 1.70 | 90 | **13.00 (±9.68)** | 11.30 | 1.45 |
| **Thromboplastin Time (sec)** | 128 | **26.87 (±8.60)** | 26.70 | 6.65 | 79 | **26.80 (±7.41)** | 26.20 | 7.50 |
| **Fibrinogen (mg/dl)** | 64 | **642.82 (±205.59)** | 604.50 | 285.33 | 36 | **360.05 (±107.39)** | 367.00 | 127.50 |
| **Sodium (mEq/l)** | 143 | **137.35 (±4.27)** | 138.00 | 5.00 | 91 | **141.32 (±2.94)** | 141.00 | 3.00 |
| **Potassium (mEq/l)** | 141 | **4.04 (±0.52)** | 4.00 | 0.70 | 91 | **4.31 (±0.41)** | 4.33 | 0.52 |
| **GOT (U/l)** | 107 | **54.03 (±45.72)** | 38.50 | 32.00 | 53 | **197.73 (±54.19)** | 190.00 | 290.00 |
| **GPT (U/l)** | 133 | **44.48 (±42.61)** | 31.00 | 26.00 | 96 | **20.06 (±8.95)** | 18.00 | 10.00 |
| **GGT (U/l)** | 89 | **99.48 (±144.58)** | 49.00 | 55.50 | 88 | **24.84 (±15.67)** | 22.00 | 14.00 |
| **LDH (U/l)** | 135 | **334.69 (±134.65)** | 304.00 | 130.00 | 73 | **212.69 (±65.19)** | 196.00 | 37.00 |

Table S3. Analytical determinations during hospital admission and at the Visit of the study.

|  | N | Median | Min-Max | Interquartile Range |
| --- | --- | --- | --- | --- |
| **MIP-1 alpha (CCL3)** | 106 | 1.06 | 0.25-16.77 | 3.32 (0.56-3.88) |
| **SDF-1 (CXCL12)** | 117 | 116.34 | 3.53-1004.37 | 79.62 (66.94-146.56) |
| **Fractalkine (CX3CL1)** | 104 | 0,74 | 0.05-38.51 | 2.63 (0.33-2.96) |
| **Eoxatin-1 (CCL11)** | 120 | 4.26 | 0.17-58.57 | 3.29 (2.88-6.17) |
| **BDNF** | 105 | 1.91 | 0.22-631.02 | 6.24 (0.52-6.76) |
| **VEGF** | 108 | 18.97 | 0.37-1705.20 | 24.82 (6.60-31.42) |
| **MCP-1 (CCL2)** | 116 | 12.30 | 0.93-189.06 | 11.31 (7.82-19.13) |
| **NFL** | 58 | 12.63 | 3.01-137.18 | 11.79 (8.03-19.81) |

Table S4. Result of plasma chemokine and Neurofilament light chain (NFL). All values are expressed in Pg/ml.

|  | Component | | | | | | | | | | |
| --- | --- | --- | --- | --- | --- | --- | --- | --- | --- | --- | --- |
|  | 1 | 2 | 3 | 4 | 5 | 6 | 7 | 8 | 9 | 10 | 11 |
| BDI |  |  |  |  |  | 0.660 |  |  |  |  |  |
| STAI State |  |  |  |  |  | 0.786 |  |  |  |  |  |
| STAI Trait |  |  |  |  |  | 0.845 |  |  |  |  |  |
| MocA. Total Score |  | 0.744 |  |  |  |  |  |  |  |  |  |
| MoCA. Executive functions. |  | 0.770 |  |  |  |  |  |  |  |  |  |
| MoCA. Animal naming |  |  |  | 0.677 |  |  |  |  |  |  |  |
| MoCA. Attention |  | 0.695 |  |  |  |  |  |  |  |  |  |
| MoCA. Language abilities |  |  |  |  |  |  |  |  |  |  |  |
| MoCA. Abstraction |  | 0.571 |  |  |  |  |  |  |  |  |  |
| MoCA. Delayed Memory |  |  |  |  |  |  |  |  |  |  |  |
| MoCA. Orientation |  |  |  | 0.570 |  |  |  |  |  |  |  |
| TAVEC Learning | 0.608 |  |  |  |  |  |  |  |  |  |  |
| TAVEC Short-term free memory | 0.670 |  |  |  |  |  |  |  |  |  |  |
| TAVEC Short Term Keys Recall | 0.832 |  |  |  |  |  |  |  |  |  |  |
| TAVEC Long-term Free Memory | 0.836 |  |  |  |  |  |  |  |  |  |  |
| TAVEC Long-Term Keys Recall | 0.828 |  |  |  |  |  |  |  |  |  |  |
| TAVEC Recognition | 0.736 |  |  |  |  |  |  |  |  |  |  |
| RCFT Copy Time |  |  |  |  |  |  |  | 0.572 |  |  |  |
| RCFT Direct Copy Score |  | 0.670 |  |  |  |  |  |  |  |  |  |
| RCFT Memory Direct Score |  | 0.656 |  |  |  |  |  |  |  |  |  |
| TMT Time A |  |  |  | -0.590 |  |  |  |  |  |  |  |
| TMT Errors A |  |  |  | -0.765 |  |  |  |  |  |  |  |
| TMT Time B |  |  |  |  |  |  |  |  |  |  |  |
| TMT Errors B |  |  |  |  |  |  | -0.554 |  |  |  |  |
| FAS-P |  |  | 0.825 |  |  |  |  |  |  |  |  |
| FAS-M |  |  | 0.803 |  |  |  |  |  |  |  |  |
| FAS-R |  |  | 0.819 |  |  |  |  |  |  |  |  |
| FAS-Animals |  |  | 0.686 |  |  |  |  |  |  |  |  |
| FAS-Vegetables | 0.553 |  | 0.549 |  |  |  |  |  |  |  |  |
| FAS-Kitchens |  |  | 0.588 |  |  |  |  |  |  |  |  |
| WAIS Direct Span |  |  |  |  |  |  |  |  |  | 0.734 |  |
| WAIS Reverse Span |  |  |  |  |  |  |  |  |  |  |  |
| FCSRT Free | 0.515 |  |  |  |  |  |  |  |  |  |  |
| FCSRT Cued |  |  |  |  | 0.739 |  |  |  |  |  |  |
| FCSRT Total |  |  |  |  | 0.508 |  |  |  |  |  |  |
| FCSRT Delayed |  |  |  |  |  |  |  |  |  |  |  |
| FCSRT Total Delayed |  |  |  |  | 0.658 |  |  |  |  |  |  |
| Length of hospital stay |  |  |  |  |  |  |  | 0.708 |  |  |  |
| Ferritin |  |  |  |  |  |  |  |  | 0.831 |  |  |
| Reactive C Protein |  |  |  |  |  |  | 0.742 |  |  |  |  |
| D-Dimer |  |  |  |  | -0.510 |  |  |  |  |  |  |

Table S5. Matrix of rotated components. Extraction method: Analysis of main components. Rotation method: Varimax normalization with Kaiser. The matrix has converged into 11 interactions.

| Component | Initial self-values | | | Sums of saturations squared from extraction | | |
| --- | --- | --- | --- | --- | --- | --- |
|  | Total | % of variance | Cumulative % | Total | % of variance | Cumulative % |
| ***1*** | ***13.332*** | ***31.743*** | ***31.743*** | ***13.332*** | ***31.743*** | ***31.743*** |
| ***2*** | ***2.745*** | ***6.535*** | ***38.278*** | ***2.745*** | ***6.535*** | ***38.278*** |
| ***3*** | ***2.121*** | ***5.051*** | ***43.329*** | ***2.121*** | ***5.051*** | ***43.329*** |
| ***4*** | ***1.998*** | ***4.758*** | ***48.087*** | ***1.998*** | ***4.758*** | ***48.087*** |
| ***5*** | ***1.547*** | ***3.684*** | ***51.771*** | ***1.547*** | ***3.684*** | ***51.771*** |
| ***6*** | ***1.499*** | ***3.568*** | ***55.339*** | ***1.499*** | ***3.568*** | ***55.339*** |
| 7 | 1.395 | 3.321 | 58.660 | 1.395 | 3.321 | 58.660 |
| 8 | 1.315 | 3.132 | 61.791 | 1.315 | 3.132 | 61.791 |
| 9 | 1.125 | 2.678 | 64.470 | 1.125 | 2.678 | 64.470 |
| 10 | 1.120 | 2.667 | 67.136 | 1.120 | 2.667 | 67.136 |
| 11 | 1.048 | 2.495 | 69.631 | 1.048 | 2.495 | 69.631 |
| 12 | 0.916 | 2.181 | 71.812 |  |  |  |
| 13 | 0.895 | 2.131 | 73.943 |  |  |  |
| 14 | 0.832 | 1.982 | 75.924 |  |  |  |
| 15 | 0.801 | 1.908 | 77.833 |  |  |  |
| 16 | 0.739 | 1.759 | 79.592 |  |  |  |
| 17 | 0.723 | 1.722 | 81.314 |  |  |  |
| 18 | 0.688 | 1.638 | 82.952 |  |  |  |
| 19 | 0.621 | 1.478 | 84.430 |  |  |  |
| 20 | 0.599 | 1.426 | 85.857 |  |  |  |
| 21 | 0.553 | 1.317 | 87.174 |  |  |  |
| 22 | 0.509 | 1.213 | 88.387 |  |  |  |
| 23 | 0.479 | 1.141 | 89.527 |  |  |  |
| 24 | 0.448 | 1.066 | 90.593 |  |  |  |
| 25 | 0.433 | 1.031 | 91.625 |  |  |  |
| 26 | 0.408 | 0.972 | 92.597 |  |  |  |
| 27 | 0.367 | 0.874 | 93.470 |  |  |  |
| 28 | 0.344 | 0.818 | 94.288 |  |  |  |
| 29 | 0.324 | 0.771 | 95.059 |  |  |  |
| 30 | 0.288 | 0.686 | 95.746 |  |  |  |
| 31 | 0.265 | 0.630 | 96.376 |  |  |  |
| 32 | 0.240 | 0.572 | 96.948 |  |  |  |
| 33 | 0.231 | 0.550 | 97.498 |  |  |  |
| 34 | 0.204 | 0.486 | 97.983 |  |  |  |
| 35 | 0.202 | 0.481 | 98.464 |  |  |  |
| 36 | 0.160 | 0.382 | 98.846 |  |  |  |
| 37 | 0.126 | 0.300 | 99.146 |  |  |  |
| 38 | 0.096 | 0.229 | 99.375 |  |  |  |
| 39 | 0.094 | 0.223 | 99.598 |  |  |  |
| 40 | 0.077 | 0.183 | 99.780 |  |  |  |
| 41 | 0.055 | 0.132 | 99.912 |  |  |  |
| 42 | 0.037 | 0.088 | 100.000 |  |  |  |

Table S6. Principal Components Analysis. Total variance explained.

| Model | | Non-standardized coefficients | | Typed coefficients | t | p |
| --- | --- | --- | --- | --- | --- | --- |
|  |  | B | Error | Beta |  |  |
|  | (Constant) | 62,936 | 1,543 |  | 40,792 | 0,000 |
|  | Component 1  (Episodic Memory) | 0,123 | 1,548 | 0,006 | 0,079 | 0,937 |
|  | ***Component 2***  ***(Global Cognition / Visuospatial abilities)*** | ***5,491*** | ***1,548*** | ***0,261*** | ***3,547*** | ***0,001*** |
|  | Component 3  (Executive functions) | 0,802 | 1,548 | 0,038 | 0,518 | 0,605 |
|  | Component 4  (Attention) | 0,800 | 1,548 | 0,038 | 0,517 | 0,606 |
|  | Component 5  (Episodic Memory) | 0,461 | 1,548 | 0,022 | 0,298 | 0,766 |
|  | ***Component 6***  ***(Depression and Anxiety Disorders)*** | ***-7,963*** | ***1,548*** | ***-0,379*** | ***-5,144*** | ***0,000*** |

Table S7 Regression analysis of the identified Principal Components. Dependent variable: EQ5 Status.

|  | | | | | | | | | | | |
| --- | --- | --- | --- | --- | --- | --- | --- | --- | --- | --- | --- |
| Model | R | R squared | Corrected R squared | Error typ. estimate | Change statistics | | | | | Durbin-Watson |  |
|  |  |  |  |  | Change in R squared | Change in F | gl1 | gl2 | Sig. Change in F |  |  |
| 1 | 0.464a | 0.215 | 0.182 | 19.022 | 0.215 | 6.613 | 6 | 145 | 0.000 | 1.678 |  |

Table S8. Model summary. Predictor variables: (Constant), Components 1, 2, 3, 4, 5 and 6. b. Dependent variable: Quality of life (EQ5 Status)

|  | | | | | | |
| --- | --- | --- | --- | --- | --- | --- |
| Model | | Sum of squares | Gl | Quadratic mean | F | p |
|  | Regression | 14355.972 | 6 | 2392.662 | 6.613 | 0.000b |
|  | Residual | 52464.454 | 145 | 361.824 |  |  |
|  | Total | 66820.426 | 151 |  |  |  |
| Table S9. ANOVAa a. Dependent variable: Quality of Life (EQ5 Status) | | | | | | |
| B. Predictor variables: (Constant), Componens 1, 2, 3, 4, 5 and 6 | | | | | | |

| Epidemiological characteristics | | | Ⅴ | NV | p |
| --- | --- | --- | --- | --- | --- |
|  | | |  |  |  |
| Sex (V. %) | | 70/152 (46.1%) | 22/40 (55%) | 48/112 (42.85%) | NS |
| Age in Years (SD) | | 69.09 (11.37) | 67.16 (11.65) | 74.76 (8.29) | P<0.005 |
| Duration of admission (Days) | | 14.38 (13.14) | 15.28 (13.86) | 11.81 (10.50) | NS |
| Ethnicity | Caucasian | 150 (98.7%) | 110 (98.21%) | 40/40 (100%) | NS |
|  | Latin | 2 (1.3%) | 2 (1.79%) | 0/0 (0%) |  |
| Level of study | Primary education | 73 (48.0%) | 54 (48.2%) | 19 (47.5%) | NS |
|  | Secondary education | 34 (22.3%) | 28 (25%) | 6 (15%) |  |
|  | Higher education | 21 (13.8%) | 12 (10.71%) | 9 (22.5%) |  |
|  | No studies | 15 (9.8%) | 12 (10.71%) | 3 (7.5%) |  |
|  | Unknown | 9 (5.9%) | 6 (5.4%) | 3 (7.5%) |  |
| Marital status | As a couple | 99 (65.1%) | 74 (66.1%) | 25 (62.5%) | NS |
|  | Single | 9 (5.9%) | 3 (7.5%) | 6 (5.4%) |  |
|  | Widower | 18 (11.8%) | 11 (9.8%) | 7 (17.5%) |  |
|  | Separate | 21 (13.8%) | 17 (15.2%) | 4 (10%) |  |
| Previous GDS | GDS1 | 108 (71.0%) | 73 (65.2%) | 35 (87.5%) | p<0.005 |
|  | GDS2 | 35 (23.0%) | 32 (28.6%) | 3 (7.5%) |  |
|  | GDS3 | 7 (4.6%) | 7 (6.3%) | 0 (0%) |  |
| Comorbidities | HT | 95 (62.5%) | 66 (58.9%) | 29 /72.5%) | NS |
|  | DM | 39 (25.6%) | 31 (27.7%) | 8 (20%) | Ns |
|  | Previous cerebrovascular disease | 47 (30.9%) | 34 (30.4%) | 13 (32.5%) | NS |
|  | Cancer disease | 8 (5.2%) | 8 (7.1%) | 0 (0%) | NS |
|  | Smoking | 15 (9.8%) | 12 (10.7%) | 3 (7.5%) | NS |
|  | Regular alcohol consumption | 28 (18.4%) | 19 (17%) | 9 (22.5%) | NS |
| General symptoms during admission | | |  |  |  |
| Fever (Temperature > 38ºC) | | 123 (80.9%) | 90 (80.4%) | 33 (82.5%) | NS |
| Cough | | 107 (69%) | 77 (68.8%) | 30 (75%) | NS |
| Dyspnea | | 90 (58.1%) | 90 (59.2%) | 22 (55%) | NS |
| Anorexia | | 47 (30.3%) | 39 (34.8%) | 8 (5.3%) | NS |
| Diarrhea | | 55 (35.5%) | 44 (39.3%) | 11 (27.5%) | NS |
| Neurological symptoms during admission | | |  |  |  |
| Headache | | 34 (21.9%) | 26(23%) | 8 (20%) | NS |
| Loss of awareness | | 11 (7.1%) | 11 (9.8%) | 0 (0%) | NS |
| Seizures | | 1 (0.6%) | 1 (0.9%) | 0 (0%) | NS |
| Stroke | | 2 (1.2%) | 2 (1.3%) | 0 (0%) | NS |
| PNS involvement | | 6 (3.9%) | 5 (4.5%) | 1 (2.5%) | NS |
| Myalgias | | 17 (11%) | 16 (14.3%) | 1 (2.5%) | P<0.05 |
| Anti-Covid19 treatments | | |  |  |  |
| Hydroxychloroquine | | 112 (72.3%) | 84 (75%) | 28 (75%) | NS |
| Lopinavir/Ritonavir | | 69 (44.5%) | 54 (48.2%) | 15 (37.5%) | NS |
| Others (Methylprednisolone. Anti-IL) | | 98 (64.4%) | 73 (65.2%) | 25 (62.5%) | NS |

Table S10. Epidemiological and clinical data during admission. **Vulnerable group (V):** People in the vulnerable group were those in whom a basic neuroinflammatory substrate or possible functional impairment of the BBB was assumed. Patients with previous diagnoses of Parkinson’s disease, amyotrophic lateral sclerosis, Multiple Sclerosis, stroke (territorial ischaemic stroke, haemorrhagic stroke, and lacunar stroke), non-lesional focal epilepsy, chronic depression, bipolar disorder, psychotic or generalized anxiety disorders. Patients with previous mild cognitive impairment with a GDS < 4 were also considered vulnerable. **Non-vulnerable group (NV)**: This group comprised those who did not meet the criteria for the vulnerable group. DM: Diabetes mellitus. GDS: Global Deterioration Scale. HT: Hypertension. PNS: Peripheral Nervous System. NS: Non-Statistical Significance.

|  | Average | SD |  | N | Average | Sd | p | Cut-off point | Abnormal (%) | | p |
| --- | --- | --- | --- | --- | --- | --- | --- | --- | --- | --- | --- |
| BDI | 14.95 | 10.73 | Ⅴ | 98 | 15.37 | 10.58 | 0.277 | 20 | 27,40% | 28.57% | 0.609 |
|  |  |  | NV | 37 | 13.92 | 11.47 |  |  |  | 24.32% |  |
| STAI State | 23.79 | 10.98 | Ⅴ | 99 | 23.65 | 11.35 | 0.148 | 25.9 (V)  29.65 (H) | 35,56% | 34.21% | 0.490 |
|  |  |  | NV | 39 | 24.19 | 10.12 |  |  |  | 39.00% |  |
| STAI Trait | 24.18 | 11.18 | Ⅴ | 100 | 23.51 | 11.33 | 0.854 | 28.73 (V)  33.83 (H) | 29,49% | 29.00% | 0.274 |
|  |  |  | NV | 39 | 24.67 | 11.91 |  |  |  | 30.76% |  |

Table S11. Results of the psychopathological assessment of the sample studied. **Vulnerable group (V):** People in the vulnerable group were those in whom a basic neuroinflammatory substrate or possible functional impairment of the BBB was assumed. Patients with previous diagnoses of Parkinson’s disease, amyotrophic lateral sclerosis, Multiple Sclerosis, stroke (territorial ischaemic stroke, haemorrhagic stroke, and lacunar stroke), non-lesional focal epilepsy, chronic depression, bipolar disorder, psychotic or generalized anxiety disorders. Patients with previous mild cognitive impairment with a GDS < 4 were also considered vulnerable. **Non-vulnerable group (NV)**: This group comprised those who did not meet the criteria for the vulnerable group.

|  | N | **Average Direct Score** | SD |  | N | Average Direct Score | SD | P |
| --- | --- | --- | --- | --- | --- | --- | --- | --- |
| **TAVEC Learning** | 118 | **35.91** | 15.02 | Ⅴ | 86 | **36.12** | 14.925 | 0.805** |
|  |  |  |  | NV | 32 | **35.34** | 15.504 |  |
| **TAVEC Short-term free memory** | 118 | **8.01** | 5.55 | Ⅴ | 85 | **8.49** | 6.148 | 0.277 |
|  |  |  |  | NV | 32 | **6.72** | 3.255 |  |
| **TAVEC Recall with short-term keys** | 118 | **8.77** | 3.74 | Ⅴ | 86 | **8.87** | 3.877 | 0.647** |
|  |  |  |  | NV | 32 | **8.50** | 3.398 |  |
| **TAVEC Long-term free memory** | 117 | **7.53** | 4.24 | Ⅴ | 85 | **7.56** | 4.360 | 0.917 |
|  |  |  |  | NV | 32 | **7.44** | 3.999 |  |
| **TAVEC Recall with long-term keys** | 117 | **8.77** | 3.87 | Ⅴ | 85 | **8.87** | 4.011 | 0.371** |
|  |  |  |  | NV | 32 | **8.50** | 3.547 |  |
| **TAVEC Recognition** | 117 | **13.36** | 3.29 | Ⅴ | 85 | **13.47** | 3.333 | 0.187 |
|  |  |  |  | NV | 32 | **13.06** | 3.202 |  |
| **BNT** | 141 | **12.30** | 3.13 | Ⅴ | 103 | **12.26** | 3.165 | 0.660 |
|  |  |  |  | NV | 38 | **15.71** | 20.933 |  |
| **RCFT Time Copy** | 123 | **242.11** | 127.66 | Ⅴ | 91 | **225.52** | 120.691 | 0.017 |
|  |  |  |  | NV | 32 | **289.31** | 136.918 |  |
| **RCFT Copy Direct Score** | 109 | **28.73** | 9.42 | Ⅴ | 81 | **28.19** | 10.041 | 0.787 |
|  |  |  |  | NV | 28 | **30.32** | 7.252 |  |
| **RCFT Memory Direct** **Score** | 101 | **11.29** | 8.47 | Ⅴ | 76 | **11.45** | 8.858 | 0.742** |
|  |  |  |  | NV | 25 | **10.80** | 7.331 |  |
| **TMT Time A (Attention)** | 114 | **94.96** | 80.83 | Ⅴ | 84 | **98.19** | 88.848 | 0.780 |
|  |  |  |  | NV | 30 | **85.90** | 52.435 |  |
| **TMT Errors A** | 108 | **0.46** | 1.23 | Ⅴ | 78 | **0.49** | 1.125 | 0.236 |
|  |  |  |  | NV | 30 | **0.40** | 1.499 |  |
| **TMT Time B (Executive Function)** | 91 | **182.25** | 141.33 | Ⅴ | 65 | **177.26** | 147.908 | 0.177 |
|  |  |  |  | NV | 26 | **194.73** | 125.216 |  |
| **TMT Errors B** | 87 | **1.87** | 2.85 | Ⅴ | 61 | **1.92** | 3.100 | 0.383 |
|  |  |  |  | NV | 26 | **1.77** | 2.233 |  |
| **FAS-P**** | 142 | **10.00** | 5.27 | Ⅴ | 104 | **9.89** | 5.381 | 0.694** |
|  |  |  |  | NV | 38 | **10.29** | 5.029 |  |
| **FAS-M**** | 142 | **8.49** | 4.92 | Ⅴ | 104 | **8.30** | 4.970 | 0.454** |
|  |  |  |  | NV | 38 | **9.00** | 4.821 |  |
| **FAS-R**** | 142 | **8.43** | 4.73 | Ⅴ | 104 | **8.13** | 4.641 | 0.220** |
|  |  |  |  | NV | 38 | **9.24** | 4.940 |  |
| **FAS Animals*** | 141 | **13.54** | 5.69 | Ⅴ | 103 | **13.68** | 5.921 | 0.631* |
|  |  |  |  | NV | 38 | **13.16** | 5.070 |  |
| **FAS Vegetables*** | 142 | **14.00** | 5.27 | Ⅴ | 104 | **14.13** | 5.429 | 0.502* |
|  |  |  |  | NV | 38 | **13.63** | 4.868 |  |
| **FAS Kitchens*** | 142 | **12.26** | 4.45 | Ⅴ | 104 | **12.17** | 4.724 | 0.582 |
|  |  |  |  | NV | 38 | **12.50** | 3.674 |  |
| **WAIS Direct Span** | 121 | **4.95** | 7.54 | Ⅴ | 91 | **5.16** | 8.659 | 0.588 |
|  |  |  |  | NV | 30 | **4.30** | 1.141 |  |
| **WAIS Reverse Span** | 121 | **3.25** | 1.386 | Ⅴ | 91 | **3.19** | 1.374 | 0.910 |
|  |  |  |  | NV | 30 | **3.43** | 1.431 |  |
| **FCSRT Free memory** | 128 | **19.53** | 9.07 | Ⅴ | 95 | **19.60** | 9.665 | 0.885** |
|  |  |  |  | NV | 33 | **19.33** | 7.231 |  |
| **FCSRT Cued memory** | 127 | **17.11** | 7.16 | Ⅴ | 94 | **16.76** | 7.524 | 0.210 |
|  |  |  |  | NV | 33 | **18.12** | 6.014 |  |
| **FCSRT Total** | 127 | **35.76** | 11.04 | Ⅴ | 94 | **35.17** | 11.847 | 0.745 |
|  |  |  |  | NV | 33 | **37.45** | 8.235 |  |
| **FCSRT Delayed** | 127 | **6.74** | 3.95 | Ⅴ | 94 | **6.67** | 4.133 | 0.738** |
|  |  |  |  | NV | 33 | **6.94** | 3.445 |  |
| **FCSRT Total Delayed** | 127 | **11.10** | 4.77 | Ⅴ | 94 | **10.74** | 4.709 | 0.500 |
|  |  |  |  | NV | 33 | **12.12** | 4.885 |  |
| **EQ-5D** | 141 | **62.94** | 21.84 | V | 103 | **61.69** | 21.28 |  |
|  |  |  |  | NV | 38 | **66.32** | 23.25 |  |

Table S12. Complete neuropsychological evaluation: . **Vulnerable group (V):** People in the vulnerable group were those in whom a basic neuroinflammatory substrate or possible functional impairment of the BBB was assumed. Patients with previous diagnoses of Parkinson’s disease, amyotrophic lateral sclerosis, Multiple Sclerosis, stroke (territorial ischaemic stroke, haemorrhagic stroke, and lacunar stroke), non-lesional focal epilepsy, chronic depression, bipolar disorder, psychotic or generalized anxiety disorders. Patients with previous mild cognitive impairment with a GDS < 4 were also considered vulnerable. **Non-vulnerable group (NV)**: This group comprised those who did not meet the criteria for the vulnerable group. Non-parametric mean comparison tests (Mann-Whitney) have been used for variables that do not meet normality criteria and parametric tests (Student T) in which if they meet them (**).

|  | N | Average | SD |  | N | Average | Z | P |
| --- | --- | --- | --- | --- | --- | --- | --- | --- |
| **MIP-1 alpha (CCL3)**  **Pg/ml** | 106 | ***2.56*** | 3.13 | Ⅴ | 80 | 2.5625 | -0.813 | 0.416 |
|  |  |  |  | NV | 26 | 2.5631 |  |  |
| **SDF-1 (CXCL12)**  **Pg/ml** | 117 | ***123.56*** | 119.65 | Ⅴ | 88 | 120.0941 | -0.451 | 0.652 |
|  |  |  |  | NV | 29 | 134.0886 |  |  |
| **Fractalkine (CX3CL1)**  **Pg/ml** | 104 | ***2.78*** | 4.81 | Ⅴ | 81 | 2.6453 | -1.155 | 0.248 |
|  |  |  |  | NV | 23 | 3.2926 |  |  |
| **Eoxatin-1 (CCL11)**  **Pg/ml** | 120 | ***6.00*** | 7.52 | Ⅴ | 90 | 6.2056 | -0.339 | 0.734 |
|  |  |  |  | NV | 30 | 5.4187 |  |  |
| **BDNF**  **Pg/ml** | 105 | ***14.60*** | 68.62 | Ⅴ | 79 | 16.6287 | -0.045 | 0.964 |
|  |  |  |  | NV | 26 | 8.4435 |  |  |
| **VEGF**  **Pg/ml** | 108 | ***65.48*** | 232.22 | Ⅴ | 78 | 61.5369 | -0.662 | 0.508 |
|  |  |  |  | NV | 30 | 75.7553 |  |  |
| **CCM-1 (CCL2)**  **Pg/ml** | 116 | ***18.54*** | 24.96 | Ⅴ | 86 | 18.3084 | -1.148 | 0.251 |
|  |  |  |  | NV | 30 | 19.2263 |  |  |
| **NFL**  **Pg/ml** | 58 | ***19.55*** | 21.47 | Ⅴ | 43 | 18.4944 | -0.613 | 0.540 |
|  |  |  |  | NV | 15 | 22.6040 |  |  |

Table S13. Result of plasma chemokine. . **Vulnerable group (V):** People in the vulnerable group were those in whom a basic neuroinflammatory substrate or possible functional impairment of the BBB was assumed. Patients with previous diagnoses of Parkinson’s disease, amyotrophic lateral sclerosis, Multiple Sclerosis, stroke (territorial ischaemic stroke, haemorrhagic stroke, and lacunar stroke), non-lesional focal epilepsy, chronic depression, bipolar disorder, psychotic or generalized anxiety disorders. Patients with previous mild cognitive impairment with a GDS < 4 were also considered vulnerable. **Non-vulnerable group (NV)**: This group comprised those who did not meet the criteria for the vulnerable group. U-Mann Whitney test. BDNF: Brain derived neurotrophic factor. MIP-1: Macrophage inflamatory proteine-1-alpha. CCL2 Chemokine (C-C motif) ligand 2. CCL3 Chemokine (C-C motif) ligand 3.  CCM-1: Cerebral cavernomatous malformation-1. NFL: Neurofilament light chain. SDF-1: Stromal cell-derived factor 1. VEGF: Vascular Endothelial Growth Factor

**Box S1: Neuropsychological evaluation method:**

The evaluation was divided into two sessions. On the first day, psychopathology and quality of life screening (FCSRT, TDB, FAS, BDI-II, STAI, NPI and EQ-5) were administered. On the second day the complete neuropsychological evaluation was carried out in this order: MoCA screening test (10 min), first part of taVEC, which includes learning, immediate free memory and with keys of verbal information (10 min). During the 25-minute TAVEC break, visuo-spatial or numerical content tasks that do not interfere with verbal episodic memory (FCR, TMT A and B and Direct and Reverse Digit Test) were administered. Then the second part of the TAVEC was made, (Free deferred memory with keys, and recognition) (10 min). The average time of both sessions was 60 minutes.
